# The new era of siRNA therapy: Advances in cancer treatment

**DOI:** 10.1002/ctm2.70363

**Published:** 2025-06-09

**Authors:** Zhuan Zhang, Miaorong Yu, Guoqing Hu, Yong Gan

**Affiliations:** ^1^ State Key Laboratory of Drug Research and Center of Pharmaceutics Shanghai Institute of Materia Medica Chinese Academy of Sciences Shanghai China; ^2^ University of Chinese Academy of Sciences Beijing China; ^3^ Department of Engineering Mechanics State Key Laboratory of Fluid Power and Mechatronic Systems Zhejiang University Hangzhou China; ^4^ NMPA Key Laboratory or Quality Research and Evaluation of Pharmaceutical Excipients National Institutes for Food and Drug Control Beijing China

## 1 DEVELOPMENT AND APPLICATION OF siRNA‐BASED THERAPEUTICS

Small interfering RNAs (siRNAs) have emerged as a groundbreaking tool in therapeutics, offering precise and efficient silencing of disease‐related genes. Through the RNA interference (RNAi) mechanism, siRNAs operate through the RNAi pathway, selectively degrading target messenger RNAs (mRNAs) and thereby preventing the synthesis of aberrant or harmful proteins.^1^ This exquisite specificity allows siRNAs to target previously ‘undruggable’ proteins, offering significant potential for treating a wide spectrum of diseases, including genetic disorders, chronic diseases, and cancers.^2^ Recent progress in siRNA technology has resulted in the clinical approval of groundbreaking therapies like Onpattro^®^, Amvuttra^®^, and Givlaari^®^, with over 130 siRNA candidates currently in clinical investigation.^3^, ^4^ These therapies have already demonstrated remarkable efficacy in conditions such as transthyretin‐mediated amyloidosis and acute hepatic porphyria, highlighting the broad therapeutic potential of this approach.

Despite these achievements, the broader application of siRNA therapeutics faces significant hurdles. Key limitations include siRNA instability in biological fluids, inefficient intracellular delivery, immunogenicity, and off‐target effects, all of which can compromise therapeutic efficacy.^5^ Overcoming these hurdles necessitates the development of innovative delivery systems that protect siRNA from degradation, promote efficient cellular uptake, and facilitate cytosolic release.^6^ Our study introduces a significant advance in siRNA‐based therapeutics through the engineering of cholesterol‐enriched exosomes (Chol/MEs).^7^ By enhancing the cholesterol content in the exosomal membrane, we created a delivery platform that enables siRNA to bypass endosomal entrapment and directly enter the cytosol via membrane fusion. This mechanism substantially improves intracellular delivery, leading to enhanced gene silencing and therapeutic efficacy. In preclinical tumour models, Chol/MEs loaded with PLK1 siRNA demonstrated superior tumour growth inhibition, reducing bioluminescence intensity to a remarkable 0.05‐fold of that observed in the PBS control group. This gene silencing efficiency significantly outperformed that of conventional transfection agents, Lipofectamine 2000 and RNAiMAX. Importantly, the biocompatibility and reduced immunogenicity of Chol/MEs address critical limitations of existing delivery technologies, offering a safer and more effective approach for siRNA‐based therapies.

## 2 EXPLOITING SYNTHETIC LETHALITY OF siRNA

Synthetic lethality has emerged as a promising strategy in cancer therapy, exploiting specific genetic vulnerabilities in tumour cells.^8^, ^9^ This approach involves the simultaneous disruption of two genes, where the inactivation of any gene alone is nonlethal, but their combined loss leads to cell death. A prime example of this is the synthetic lethal interaction between BRCA1/2 mutations and PARP inhibitors.^10^ This strategy selectively targets cancer cells with deficiencies in DNA repair, while sparing healthy cells.

**FIGURE 1:**
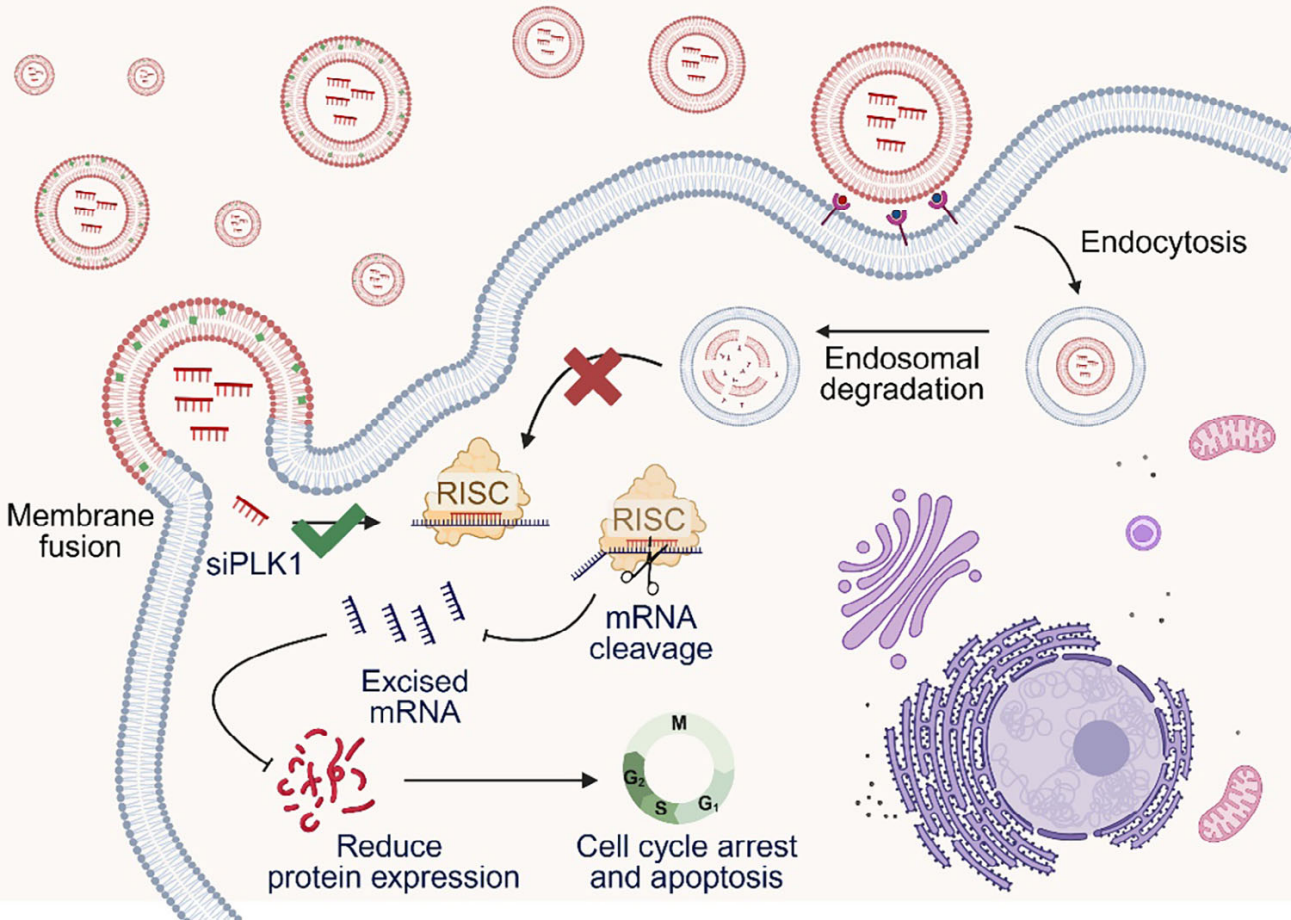
Schematic illustration of siPLK1 treatment of tumours. Delivery of siPLK1 into tumour cells via Chol/ME‐mediated membrane fusion leads to a significant increase in G2/M phase arrest and apoptosis in HCT116 colorectal cancer cells. Created with BioRender.com.

siRNA offers a potent means of inducing synthetic lethality by precisely silencing genes essential for tumour survival. Recent advances in RNAi and CRISPR‐based screening have identified numerous synthetic lethal targets, including ATR, CDK1, and PLK1, all of which play critical roles in cancer cell proliferation.^11^, ^12^, ^13^ Our study focuses on PLK1, a key regulator of mitosis, as a synthetic lethality target (Figure 1). Delivery of siPLK1 via Chol/MEs resulted in a significant increase in G2/M phase arrest and apoptosis in HCT116 colorectal cancer cells.^7^ This strategy effectively suppresses tumour growth by exploiting the dependency of cancer cells on PLK1 for survival.

The combination of siRNA's precision targeting with the concept of synthetic lethality provides a novel and highly targeted therapeutic strategy for cancer treatment. This innovation not only enhances the efficacy of siRNA‐based therapies but also broadens their applicability to a wide range of cancers with distinct genetic profiles.

## 3 ORAL DELIVERY OF siRNA THERAPEUTICS

Oral administration represents the most patient‐friendly and widely accepted route of drug delivery, offering non‐invasive and convenient treatment option. However, oral delivery of siRNA therapeutics presents a formidable challenge due to enzymatic degradation within the gastrointestinal (GI) tract, poor mucosal permeability, and limited systemic absorption.^14^ Overcoming these barriers is essential to realise the full clinical potential of siRNA therapies.^15^

Oral siRNA therapy holds unique clinical advantages, including improved patient compliance, particularly for chronic diseases requiring long‐term treatment, and the potential for reduced healthcare costs. Although no oral siRNA therapies have yet been approved for clinical trials, recent advances in delivery technologies have brought this goal closer to reality. Preclinical studies, such as Alnylam's GalNAc‐conjugated siRNA formulation combined with permeation enhancers, have shown efficacy comparable to that of subcutaneous injection.^16^

**FIGURE 2:**
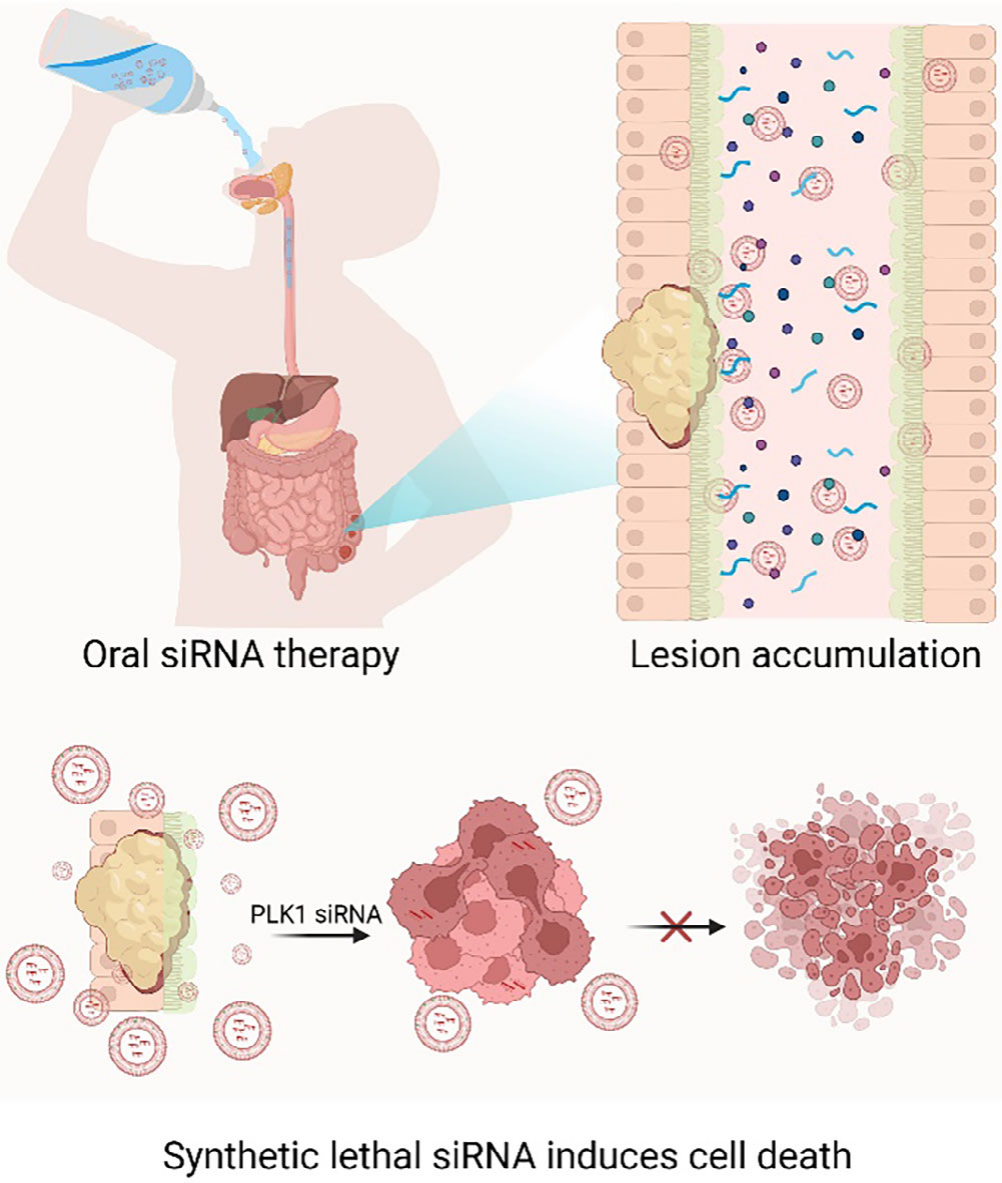
Schematic of Chol/MEs‐mediated oral siRNA delivery and therapeutic efficacy. Chol/MEs protect siRNA from degradation, enhance mucosal penetration, and facilitate direct cytosolic delivery via membrane fusion. In colorectal cancer models, oral administration of PLK1 siRNA‐loaded Chol/MEs significantly inhibits tumour growth, demonstrating their potential for noninvasive siRNA therapy. Created with BioRender.com.

Our study demonstrates a breakthrough in this field, showcasing the successful oral delivery of siRNA using Chol/MEs (Figure 2). These engineered exosomes effectively protect siRNA from degradation within the GI tract and facilitate mucosal penetration. By enabling direct cytosolic delivery through membrane fusion, Chol/MEs overcome the major barriers to oral siRNA therapy. In preclinical models of colorectal cancer, oral administration of Chol/MEs loaded with PLK1 siRNA led to substantial tumour growth inhibition. This achievement underscores the potential of Chol/MEs as a versatile platform for noninvasive siRNA delivery, addressing unmet needs in both cancer therapy and chronic disease management.

## 4 CONCLUSION

The evolution of nucleic acid drugs has brought us to the forefront of precision medicine, providing transformative solutions for cancer and other challenging diseases. By addressing key barriers in delivery, our work with cholesterol‐enriched exosomes advances the therapeutic potential of siRNA, particularly in the context of exploiting synthetic lethality and enabling oral administration. These innovations represent a significant step forward in the clinical translation of siRNA therapies, paving the way for safer, more effective, and accessible treatments in the new era of RNAi‐based medicine.

## AUTHOR CONTRIBUTIONS

Z.Z. drafted the manuscript. M.Y., G.H., and Y.G. contributed to the revision. All authors reviewed and approved the final manuscript.

## CONFLICT OF INTEREST STATEMENT

The authors declare no competing interests.
